# Long noncoding RNA XIST knockdown relieves the injury of microglia cells after spinal cord injury by sponging miR-219-5p

**DOI:** 10.1515/med-2021-0292

**Published:** 2021-08-03

**Authors:** Xueren Zhong, Yongzheng Bao, Qiang Wu, Xinhua Xi, Wengang Zhu, Sanmei Chen, Junjian Liao

**Affiliations:** Department of Spine Surgery, Yue Bei People’s Hospital, Shaoguan City 512026, Guangdong Province, China; Department of Arthrology, Yue Bei People’s Hospital, Shaoguan City, Guangdong Province, China; Department of Emergency, Yue Bei People’s Hospital, Shaoguan City, Guangdong Province, China; Department of Trauma Orthopedic, Yuebei People’s Hospital Affiliated to Shantou University Medical College, Shaoguan City, Guangdong Province, China

**Keywords:** XIST, miR-219-5p, NF-κB pathway, SCI, LPS, BV2 cells

## Abstract

Long noncoding RNAs have been demonstrated to play crucial roles in the pathogenesis of spinal cord injury (SCI). In this study, we aimed to explore the roles and underlying mechanisms of lncRNA X-inactive specific transcript (XIST) in SCI progression. SCI mice model was constructed and evaluated by the Basso–Beattie–Bresnahan method. The SCI cell model was constructed by treating BV2 cells with lipopolysaccharide (LPS). The levels of XIST and miR-219-5p were determined by the reverse transcription quantitative polymerase chain reaction. The concentrations of inflammatory cytokines were measured by enzyme-linked immunosorbent assay. Protein levels were measured via western blot assay. Cell viability and apoptosis were evaluated by cell counting kit-8 assay and flow cytometry analysis, respectively. The relationship between XIST and miR-219-5p was analyzed by online tool starBase, dual-luciferase reporter assay, and RNA immunoprecipitation assay. As a result, the XIST level was enhanced and the miR-219-5p level was declined in the SCI mice model. XIST was also upregulated in LPS-induced BV2 cells. LPS treatment restrained BV2 cell viability and accelerated apoptosis and inflammatory response. XIST knockdown effectively weakened LPS-induced BV2 cell injury. miR-219-5p was identified as a target of XIST. Moreover, inhibition of miR-219-5p restored the impacts of XIST knockdown on cell viability, apoptosis, and inflammation in LPS-treated BV2 cells. In addition, LPS-induced XIST promoted the activation of the nuclear factor-κB (NF-κB) pathway by sponging miR-219-5p. In conclusion, XIST silencing promoted microglial cell viability and repressed apoptosis and inflammation by sponging miR-219-5p, thus promoting the recovery of SCI.

## 1 Introduction

Spinal cord injury (SCI) is one of the most serious types of nerve injury caused by external direct or indirect factors [8,18]. The prognosis of patients with SCI is extremely dismal, causing limb movement disorders, loss of cognitive function, and even paralysis, which seriously affect people’s quality of life [9,21]. Currently, although a large number of studies have explored the treatment strategies for SCI, the effects remain unsatisfactory [1,4]. Thus, it is crucial to explore the potential mechanisms of SCI development and develop novel therapeutic targets for SCI.

Long noncoding RNAs (lncRNAs) are a series of noncoding RNAs (ncRNAs) containing >200 nucleotides (nts) in length, exerting their functions mainly by sponging microRNAs (miRNAs) [19,25]. It has been demonstrated that lncRNAs regulate a variety of physiological functions, and neurological diseases, including SCI, have been demonstrated [24]. For example, Zheng et al. disclosed that the elevation of taurine upregulated gene type 1 (TUG1) repressed lipopolysaccharide (LPS)-stimulated PC-12 cell damage, as demonstrated by the promotion in cell viability and the suppression in cell apoptosis and inflammation, by decreasing miR-127 and inactivating nuclear factor-κB (NF-κB) pathway [30]. Zhou et al. claimed that metastasis-associated lung adenocarcinoma transcript 1 (MALAT1) level was conspicuously raised in SCI mice and LPS-activated microglial cells, and MALAT1 knockdown relieved LPS-stimulated inflammatory injury by regulating miR-199b and IκB kinase β (IKKβ)/NF-κB pathway [31]. These reports indicated the vital roles of lncRNAs in SCI development. As for X-inactive specific transcript (XIST), Zhao et al. uncovered that XIST deficiency facilitated the recovery of SCI by reducing miR-27a and elevating Smurf1 [29]. Nevertheless, the molecular mechanisms of XIST in regulating SCI progression are not well understood.

miRNAs are small ncRNAs consisting of ∼22 nts and exert vital regulatory roles at the posttranscriptional level [6]. An increasing number of miRNAs have been identified to be closely linked to the progression of SCI. For instance, miR-27a-3p inhibited the inflammatory injury of SCI by interacting with toll-like receptor 4 (TLR4) [28]. miR-129-5p repressed the inflammatory response and apoptosis in LPS-stimulated BV2 cells via the modulation of high-mobility group protein B1 (HMGB1)/TLR4/NF-κB pathway [22]. More importantly, a previous study by Zhu et al. showed that miR-219-5p was able to promote the recovery of SCI and motor function by regulating inflammation and oxidative stress [32]. However, the exact roles of miR-219-5p in SCI are largely unknown. Through online tool starBase, we found that the XIST contained potential binding sites with miR-219-5p. However, the relationship between XIST and miR-219-5p in regulating SCI progression has not been explored.

In this research, we established SCI mice and cell models and explored the effects of XIST on BV2 cell viability, apoptosis, and inflammation after SCI. Moreover, the possible mechanism and signaling pathway of XIST in SCI progression were further investigated.

## 2 Materials and methods

### 2.1 Construction of SCI mice model

Adult C57bl/6J mice (female; 20–25 g) were purchased from the Vital River (Beijing, China) and divided into two groups: SCI group (*n* = 10) and sham group (*n* = 10). In SCI groups, the mice were incised along the neck after anesthesia, and then C5 lamina was excised to exhibit the dural sac. Next, adjust the hammer position of the spinal impactor on the C5 spinal cord. After that, bleeding was stopped and the incision layer by layer was sutured. In the sham group, C5 lamina was removed in the mice and C5 spinal cord was not impinged. The hindlimb locomotor activity of the mice was evaluated by Basso–Beattie–Bresnahan (BBB) Locomotor Rating Scale score. Subsequently, after 7 days of SCI, the mice were anesthetized and the spinal cord tissue specimens were collected for the following experiments. The study was allowed by the Ethics Committee of Animal Research of Yue Bei People’s Hospital and conducted according to the Guidelines for Care and Use of Laboratory Animals of “National Institutes of Health.”

### 2.2 Cell culture and treatment

The murine microglial cells (BV2) were bought from Procell (Wuhan, China) and cultured in Dulbecco’s modified Eagle’s medium (DMEM; Gibco, Grand Island, NY, USA) added with 10% fetal bovine serum (FBS; Gibco) and 1% penicillin–streptomycin (Gibco) in an incubator containing 5% CO_2_ at 37°C.

For LPS treatment, BV2 cells were cultured with LPS (1, 10, 100, or 1,000 ng/mL; Solarbio, Beijing, China) for 24 h. For the SCI cell model, BV2 cells were activated with 100 ng/mL LPS for 24 h.

### 2.3 Cell transfection

XIST small-interfering RNA (si-XIST) and scrambled siRNA control (si-NC), the overexpression vector of XIST and its control (lnc-NC), miR-219-5p mimics (miR-219-5p) and its control (NC), miR-219-5p inhibitors (anti-miR-219-5p), and anti-NC were synthesized by GeneCopoeia (Guangzhou, China) and then transfected into BV2 cells with Lipofectamine 2000 (Invitrogen, Carlsbad, CA, USA). After 6 h of transfection, BV2 cells were triggered with 100 ng/mL LPS (Solarbio).

### 2.4 Reverse transcription quantitative polymerase chain reaction (RT-qPCR)

Total RNA in spinal cord tissues and BV2 cells was isolated utilizing TRIzol (Invitrogen). Next, the RNAs were reversely transcribed into complementary DNAs (cDNAs) utilizing miRNA 1st Strand cDNA Synthesis Kit (Vazyme, Nanjing, China) or avian myeloblastosis virus (AMV) reverse transcriptase (Promega, Madison, WI, USA). Afterward, RT-qPCR reaction was manipulated utilizing AceQ Universal SYBR qPCR Master Mix (Vazyme) and specific primers (GeneCopoeia) on an ABI 7500 PCR system (Applied Biosystems, Foster City, CA, USA). The primers were as follows: XIST: (F: 5′-CGGGTCTCTTCAAGGACATTTAGCC-3′ and R: 5′-GCACCAATACAGAGGAATGGAGGG-3′); miR-219-5p: (F: 5′-ACACTCCAGCTGGGTGATTGTCCAAACGCAAT-3′ and R: 5′-CTCAACTGGTGTCGTGGAGTCGGC-3′); glyceraldehyde 3-phosphate dehydrogenase (GAPDH): (F: 5′-GAAGATGGTGATGGGATTTC-3′ and R: 5′-GAAGGTGAAGGTCGGAGT-3′); U6: (F: 5′-CTCGCTTCGGCAGCACA-3′ and R: 5′-AACGCTTCACGAATTTGCGT-3′). Glyceraldehyde 3-phosphate dehydrogenase (GAPDH) or U6 served as the internal reference. The expression was computed via the 2^−ΔΔCt^ strategy.

### 2.5 Enzyme-linked immunosorbent assay (ELISA)

The concentrations of inflammatory cytokines (TNF-α, IL-1β, IL-6, and IL-10) in the spinal cord tissue extracts or BV2 cell supernatants were measured by ELISA kits (ab208348; ab197742; ab100713; ab108870; Abcam, Cambridge, MA, USA) based on the guidelines of manufacturers. The absorbance was measured at 450 nm utilizing a microplate reader (Bio-Rad, Hercules, CA, USA), and the concentrations were calculated based on the standard curve.

### 2.6 Western blot assay

Protein isolation was done using radioimmunoprecipitation assay buffer (CWBio, Beijing, China), and protein concentration was detected using a bicinchoninic acid protein assay kit (Tiangen, Beijing, China). Then, an equal amount of proteins was split by sodium dodecyl sulfonate-polyacrylamide gel (Solarbio) electrophoresis and blotted onto polyvinylidene difluoride membranes (Millipore, Billerica, MA, USA). The membranes were blocked using 5% nonfat milk for 1 h at indoor temperature. Next, the membranes were immunoblotted with primary antibodies against GAPDH (ab181602; Abcam), total p65 (t-p65; ab16502; Abcam), phosphorylated p65 (p-p65; ab86299; Abcam), B-cell lymphoma-2 (Bcl-2, ab196495; Abcam), BCL2-associated X (Bax, ab180733; Abcam), cleaved-caspase 3 (C-caspase 3, ab49822; Abcam), or total-caspase 3 (t-caspase 3, ab90437; Abcam) overnight at 4°C and then incubated with corresponding secondary antibody (ab6789; Abcam) for 1.5 h at indoor temperature. Finally, the protein bands were exposed through an enhanced chemiluminescence reagent (Vazyme) and analyzed by software Image J.

### 2.7 Cell counting kit-8 (CCK-8) assay

After treatment with LPS and transfection, BV2 cells were harvested to assess cell viability through CCK-8 assay. Briefly, BV2 cells were seeded into 96-well plates and cultivated for 24 h. Next, 10 μL CCK-8 (Beyotime, Shanghai, China) was added into each well with incubation for another 4 h at room temperature. The optical density (OD) value (at 450 nm) was recorded using a microplate reader (Bio-Rad).

### 2.8 Flow cytometry analysis

After treated with 100 ng/mL LPS for 24 h or transfected with indicated synthetic plasmids or oligonucleotides followed by LPS treatment for 24 h, BV2 cells were harvested and Annexin V-fluorescein isothiocyanate (FITC) Apoptosis Detection Kit (Vazyme) was utilized for the analysis of cell apoptosis. In brief, the harvested BV2 cells were rinsed with cold PBS (Sangon, Shanghai, China) and resuspended in binding buffer. BV2 cells were mixed with 5 µL Annexin V-FITC and 5 µL propidium iodide (PI) for 15 min in the dark at indoor temperature. Finally, the apoptotic cells were estimated with flow cytometry (Beckman Coulter, Atlanta, GA, USA).

### 2.9 Dual-luciferase reporter assay

The fragments of XIST including the wild-type (wt) or mutant (mut) binding sites of miR-219-5p were cloned into psi-CHECK2 plasmid (Promega), generating XIST-wt and XIST-mut, respectively. BV2 cells were subsequently plated into six-well plates and transfected with XIST-wt (or XIST-mut) and miR-219-5p (or NC, anti-miR-219-5p, anti-NC). The renilla and firefly luciferase activities were detected using a Dual-Luciferase Reporter Assay System (Promega) after 48 h of co-transfection.

### 2.10 RNA immunoprecipitation (RIP) assay

Magna RNA-binding protein immunoprecipitation kit (Millipore) was exploited for RIP assay. Briefly, BV2 cells were disrupted in RIP buffer, and cell extracts were cultivated with magnetic beads, which were conjugated with anti-immunoglobulin G (anti-IgG) or anti-argonaute-2 (anti-Ago2). Then, the immunoprecipitated RNAs were purified, and the enrichment of XIST and miR-219-5p was examined via RT-qPCR analysis.

### 2.11 Statistical analysis

All experiments were manipulated in triplicate. Data analysis was executed using the software GraphPad Prism 7, and the results were exhibited as mean ± standard deviation. The differences between two sets were estimated by Student’s *t*-test, whereas differences among three groups were estimated by one-way analysis of variance followed by Tukey’s test. It was defined as significant if *P* < 0.05.


**Ethics approval and consent to participate:** The hospital’s Institutional Review Board approved the current study.

## 3 Results

### 3.1 XIST was upregulated and miR-219-5p was downregulated in SCI mice model

**Figure 1:**
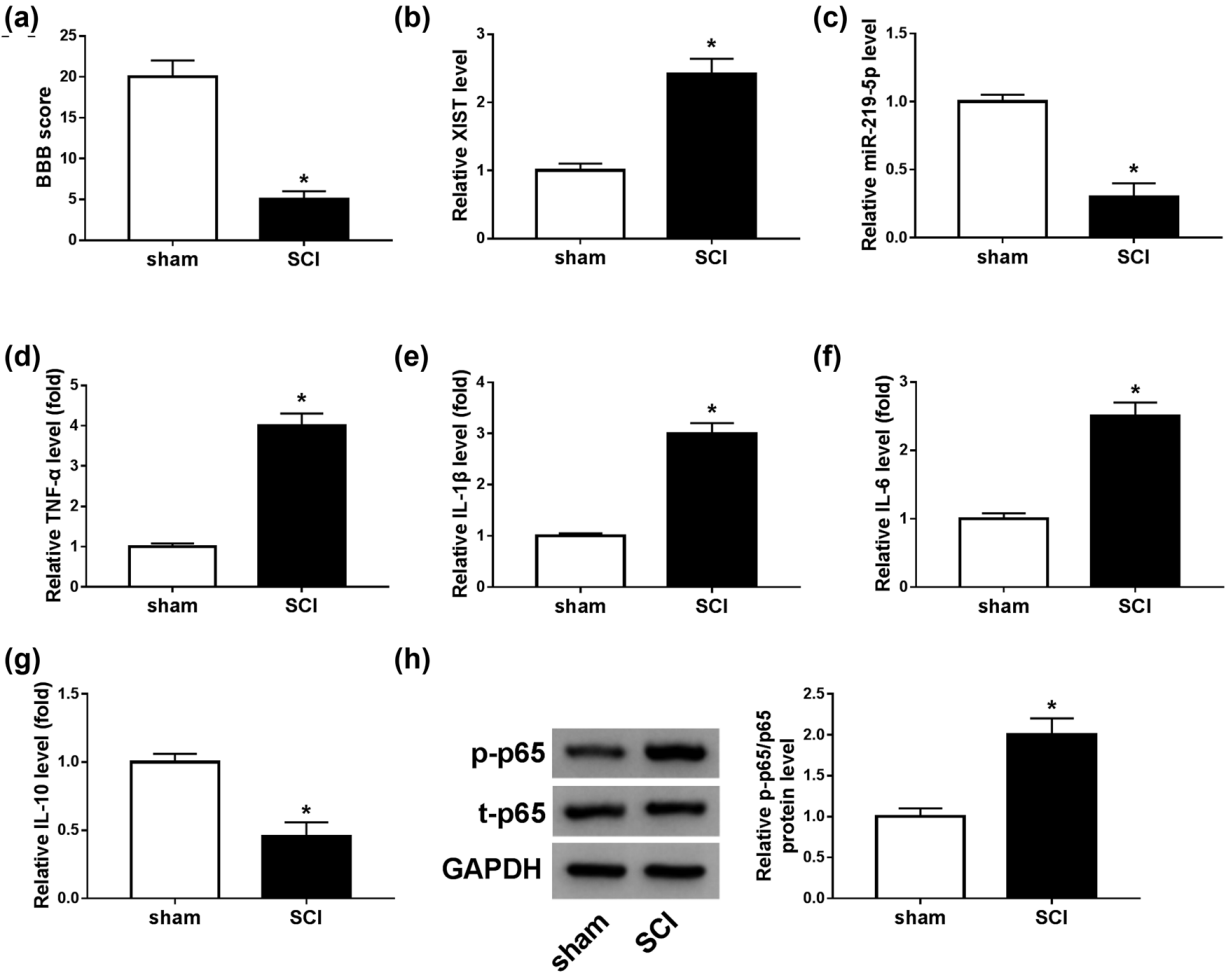
XIST was elevated and miR-219-5p was reduced in SCI mice model. (a) The BBB score method was used to evaluate the locomotor function changes of mice in SCI and sham groups. (b and c) RT-qPCR analysis was performed for the expression levels of XIST and miR-219-5p in the spinal cord tissues from SCI and sham groups. (d–g) ELISA assay was conducted to determine the levels of TNF-α, IL-1β, IL-6, and IL-10 in the spinal cord tissue extracts of SCI groups and sham groups. (h) Western blot assay was conducted for the protein levels of p-p65 and t-p65 in the spinal cord tissues from SCI and sham groups. *P < 0.05.

After the mice model of SCI was established, the recovery of motor function in mice was evaluated by the BBB method. The results showed that the hindlimb locomotor activity was markedly decreased after spinal cord contusions, as indicated by the decreased BBB score in SCI groups compared with sham operation groups (Figure 1a). The results suggested that the SCI mice model was successfully established. Then, we determined the expression levels of XIST and miR-219-5p in the spinal cord tissues from SCI groups and sham groups by RT-qPCR analysis. The results showed that the XIST level was notably elevated and miR-219-5p was conspicuously reduced in the spinal cord tissues from SCI groups compared with sham groups (Figure 1b and c). Next, we detected the levels of inflammatory cytokines (including TNF-α, IL-1β, IL-6, and IL-10) in SCI mice through ELISA. As shown in Figure 1d–g, the levels of TNF-α, IL-1β, and IL-6 were drastically elevated and the level of IL-10 was distinctly declined in SCI groups compared to sham operation groups. Herein, SCI also caused a noteworthy elevation in p-p65 protein level compared to sham groups (Figure 1h). Collectively, XIST was abnormally increased and miR-219-5p was abnormally decreased in the SCI mice model.

### 3.2 LPS repressed cell viability and induced apoptosis and inflammatory response in BV2 cells

**Figure 2:**
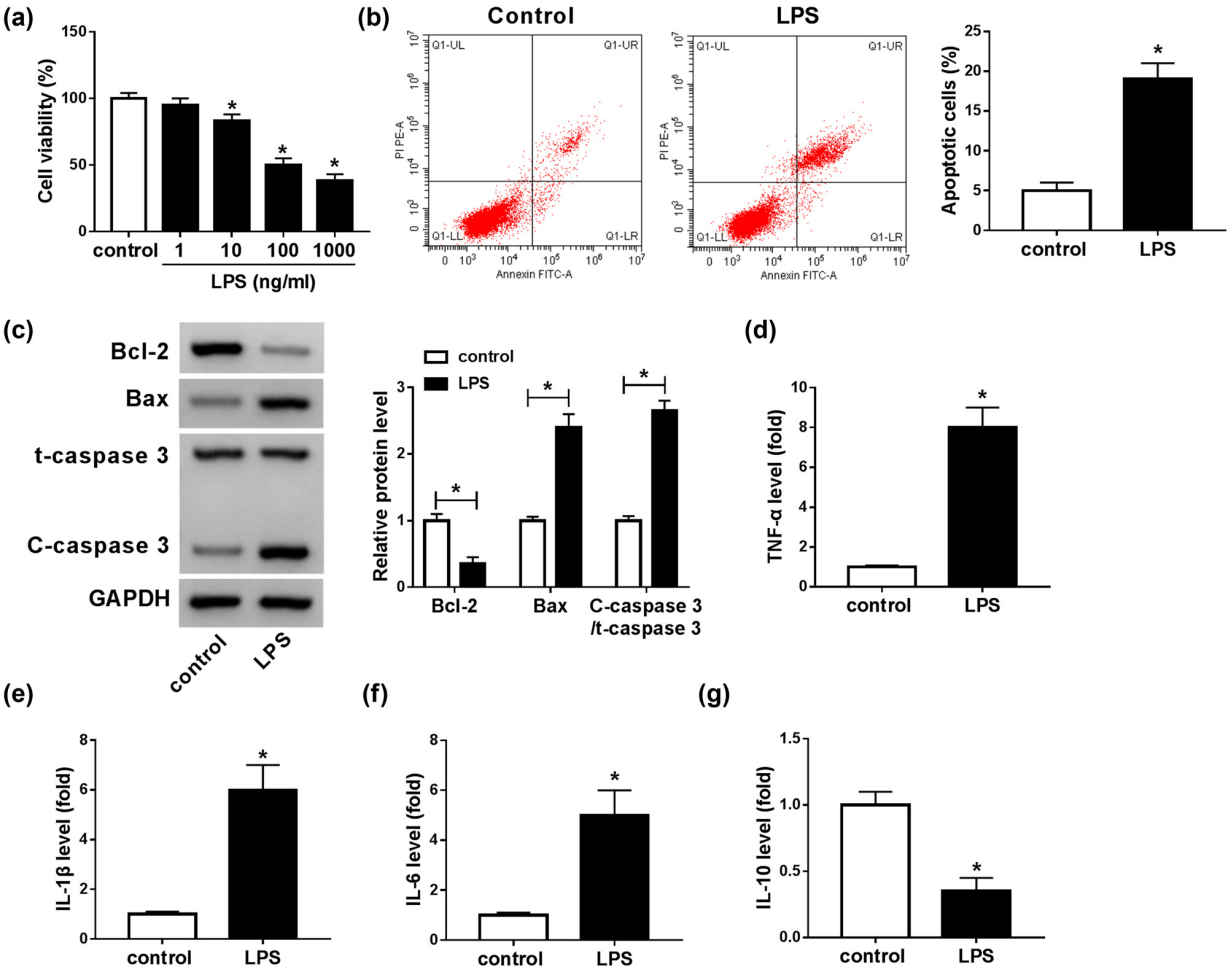
LPS treatment suppressed BV2 cell viability and promoted apoptosis and inflammatory damage. (a) BV2 cells were treated with LPS (1, 10, 100, and 1,000 ng/mL) for 24 h, and then, the viability of BV2 cells was evaluated by CCK-8 assay. (b) The apoptosis of BV2 cells treated with 100 ng/mL LPS was investigated by flow cytometry analysis. (c) The protein levels of Bcl-2, Bax, t-caspase 3, and C-caspase 3 in 100 ng/mL LPS-treated BV2 cells were measured through western blot assay. (d–g) The levels of TNF-α, IL-1β, IL-6, and IL-10 were measured by ELISA in LPS-stimulated BV2 cells. *P < 0.05.

LPS-induced microglial cell is a commonly used SCI model *in vitro*. To establish the SCI model *in vitro*, microglial cells (BV2) were exposed to different concentrations of LPS (1, 10, 100, and 1,000 ng/mL) for 24 h. As illustrated by the CCK-8 assay, the viability of BV2 cells was markedly repressed by LPS in a dose-dependent manner (Figure 2a). There was no significant difference in BV cell viability between 100 ng/mL groups and 1,000 ng/mL groups; thus, 100 ng/mL LPS was utilized in the following study. Flow cytometry analysis showed that the apoptosis of BV2 cells was promoted by 100 ng/mL LPS treatment (Figure 2b). Meanwhile, we determined the levels of apoptosis-related proteins (Bcl-2, Bax, and C-caspase 3) in 100 ng/mL LPS-stimulated BV2 cells by western blot assay. The results indicated that LPS treatment led to an apparent reduction in Bcl-2 expression and an obvious elevation in Bax and C-caspase 3 expression in BV2 cells compared to control groups (Figure 2c). In addition, ELISA results showed that LPS distinctly enhanced the levels of TNF-α, IL-1β, and IL-6 and reduced the level of IL-10 in BV cells compared to control groups (Figure 2d–g). These observations suggested that LPS-induced SCI cell model was successfully constructed *in vitro*.

### 3.3 XIST knockdown abrogated LPS-mediated BV2 cell viability, apoptosis, and inflammatory response

**Figure 3:**
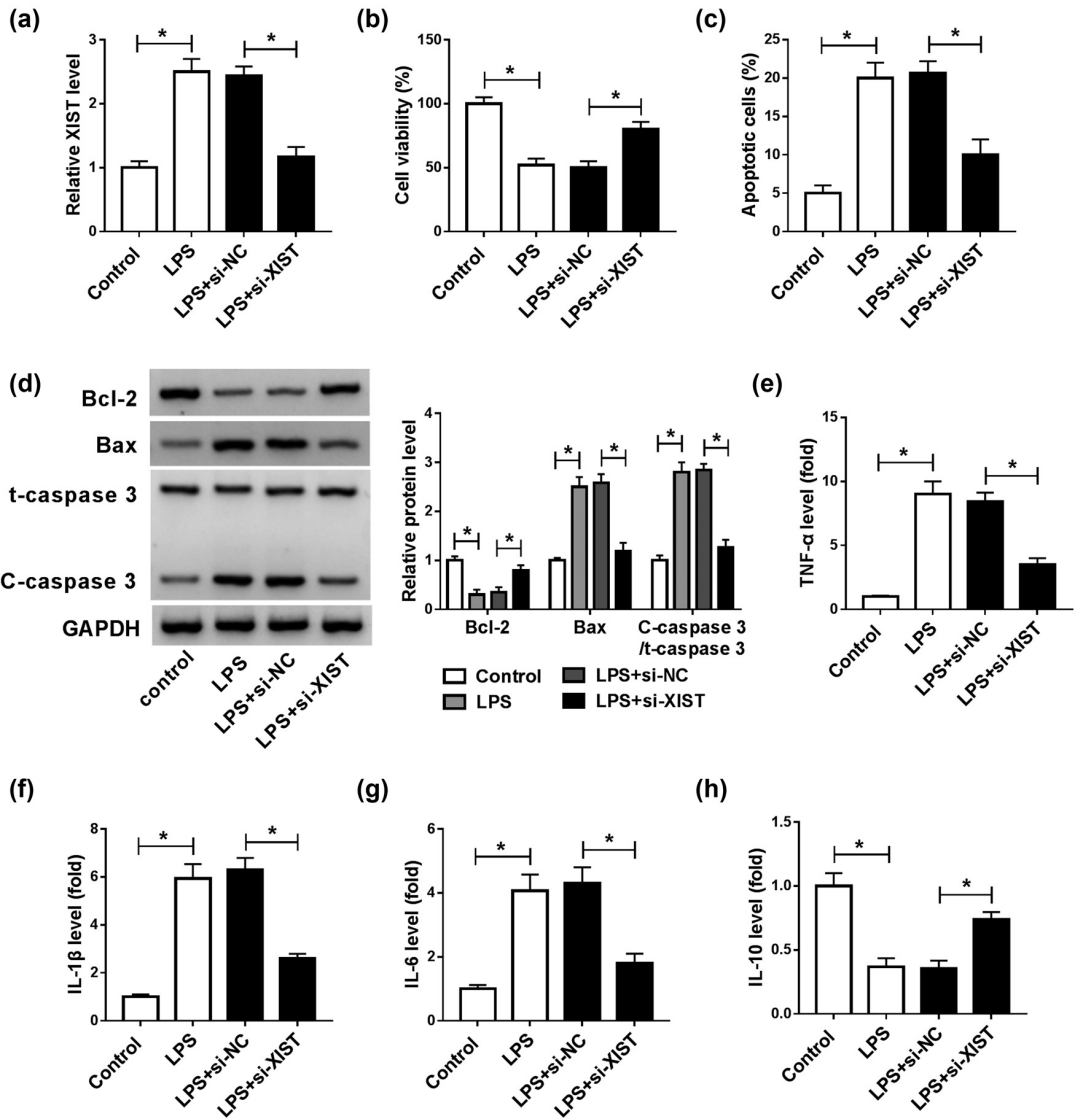
XIST silencing restored the impacts of LPS on cell viability, apoptosis, and inflammation in BV2 cells. BV2 cells were transfected with si-XIST or si-NC and then treated with 100 ng/mL LPS. (a) The expression level of XIST in BV2 cells was determined by the RT-qPCR assay. (b and c) The viability and apoptosis of BV2 cells were evaluated through CCK-8 assay and flow cytometry analysis. (d) The protein levels of Bcl-2, Bax, t-caspase 3, and C-caspase 3 in BV2 cells were examined by western blot assay. (e–h) The secretion of TNF-α, IL-1β, IL-6, and IL-10 in BV2 cells was detected by ELISA. *P < 0.05.

As shown in Figure 3a, the XIST level was increased in LPS-stimulated BV2 cells, indicating that XIST might be involved in the regulation of LPS-mediated microglial cell viability, apoptosis, and inflammatory cytokine production. Thus, we explored the function of XIST in LPS-stimulated microglial cell progression by transfecting si-XIST or si-NC into BV2 cells and then treating the transfected cells with LPS. As demonstrated by the RT-qPCR assay, XIST silencing markedly suppressed the XIST level in LPS-induced BV2 cells (Figure 3a). CCK-8 assay showed that the inhibitory effect on cell viability mediated by LPS was restored by decreasing XIST expression in BV2 cells (Figure 3b). Flow cytometry analysis indicated that LPS-induced cell apoptosis was repressed by XIST knockdown in BV2 cells (Figure 3c). Moreover, western blot assay results showed that LPS treatment suppressed Bcl-2 expression and promoted Bax and C-caspase 3 expression in BV2 cells, while XIST knockdown effectively restored the impacts (Figure 3d). In addition, we observed that the upregulation of TNF-α, IL-1β, and IL-6 and the downregulation of IL-10 mediated by LPS were restored by reducing XIST expression in BV2 cells (Figure 3e–h). To sum up, XIST knockdown could relieve LPS-induced injury in BV2 cells.

### 3.4 XIST negatively regulated miR-219-5p expression by directly targeting

**Figure 4:**
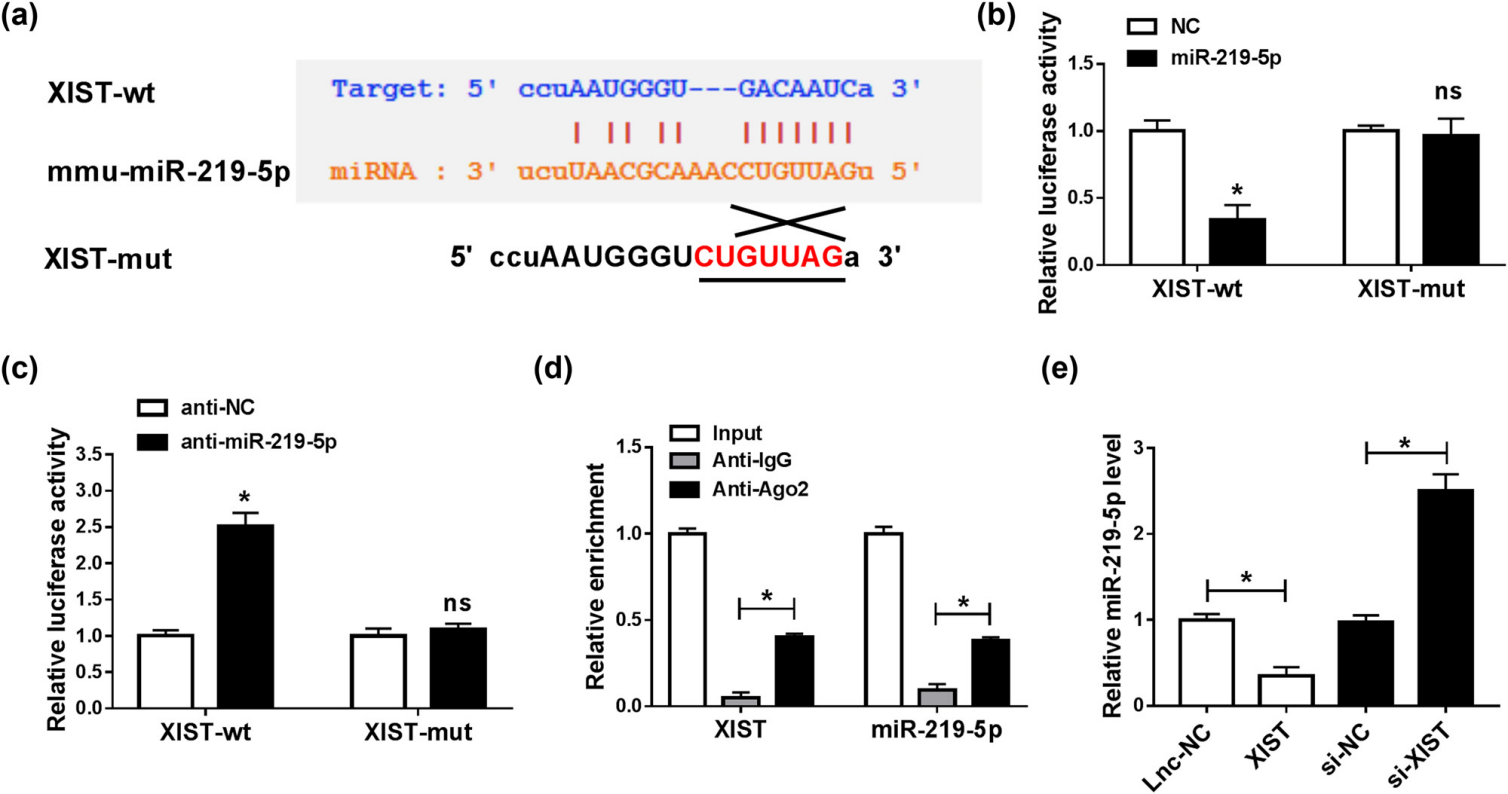
XIST sponged miR-219-5p to suppress miR-219-5p expression. (a) The potential binding sites between XIST and miR-219-5p were predicted by starBase. (b and c) XIST-wt (or XIST-mut) and miR-219-5p (or NC, anti-miR-219-5p, anti-NC) were co-transfected into BV2 cells, and then, dual-luciferase reporter assay was conducted to measure the luciferase activity in BV2 cells. (d) After RIP assay, the abundance of XIST and miR-219-5p in anti-IgG or anti-Ago2 immunoprecipitates in BV2 cells was measured by RT-qPCR analysis. (e) The expression level of miR-219-5p in BV2 cells transfected with lnc-NC, XIST, si-NC, or si-XIST was determined using the RT-qPCR assay. *P < 0.05.

To explore the potential mechanism of XIST regulating BV2 cell progression, we analyzed the targets of XIST through online tool starBase (http://starbase.sysu.edu.cn/agoClipRNA.php?source=lncRNA&flag=target&clade=mammal&genome=mouse&assembly=mm10&miRNA=all&clipNum=1&deNum=0&target=Xist). The results displayed that miR-219-5p contained the complementary sequences of XIST (Figure 4a). Then dual-luciferase reporter assay and RIP assay were carried out to confirm the interaction between miR-219-5p and XIST. As suggested by the dual-luciferase reporter assay, miR-219-5p transfection markedly inhibited the luciferase activity of XIST-wt and anti-miR-219-5p transfection conspicuously elevated the luciferase activity of XIST-wt in BV2 cells, while the luciferase activity of XIST-mut was not affected by miR-219-5p or anti-miR-219-5p (Figure 4b and c). The results of the RIP assay showed that the levels of XIST and miR-219-5p were all enriched in anti-Ago2 protein complexes in BV2 cells compared to anti-IgG control groups, further confirming the interaction between XIST and miR-219-5p (Figure 4d). Thereafter, we explored the effect of XIST on miR-219-5p expression by transfecting XIST or si-XIST into BV2 cells. Our results showed that XIST transfection apparently decreased miR-219-5p level in BV2 cells, while si-XIST transfection exhibited the opposite results (Figure 4e). These observations indicated that XIST could negatively modulate miR-219-5p expression by direct interaction.

### 3.5 miR-219-5p inhibition reversed the effects of XIST knockdown on cell viability, apoptosis, and inflammation in LPS-stimulated BV2 cells

**Figure 5:**
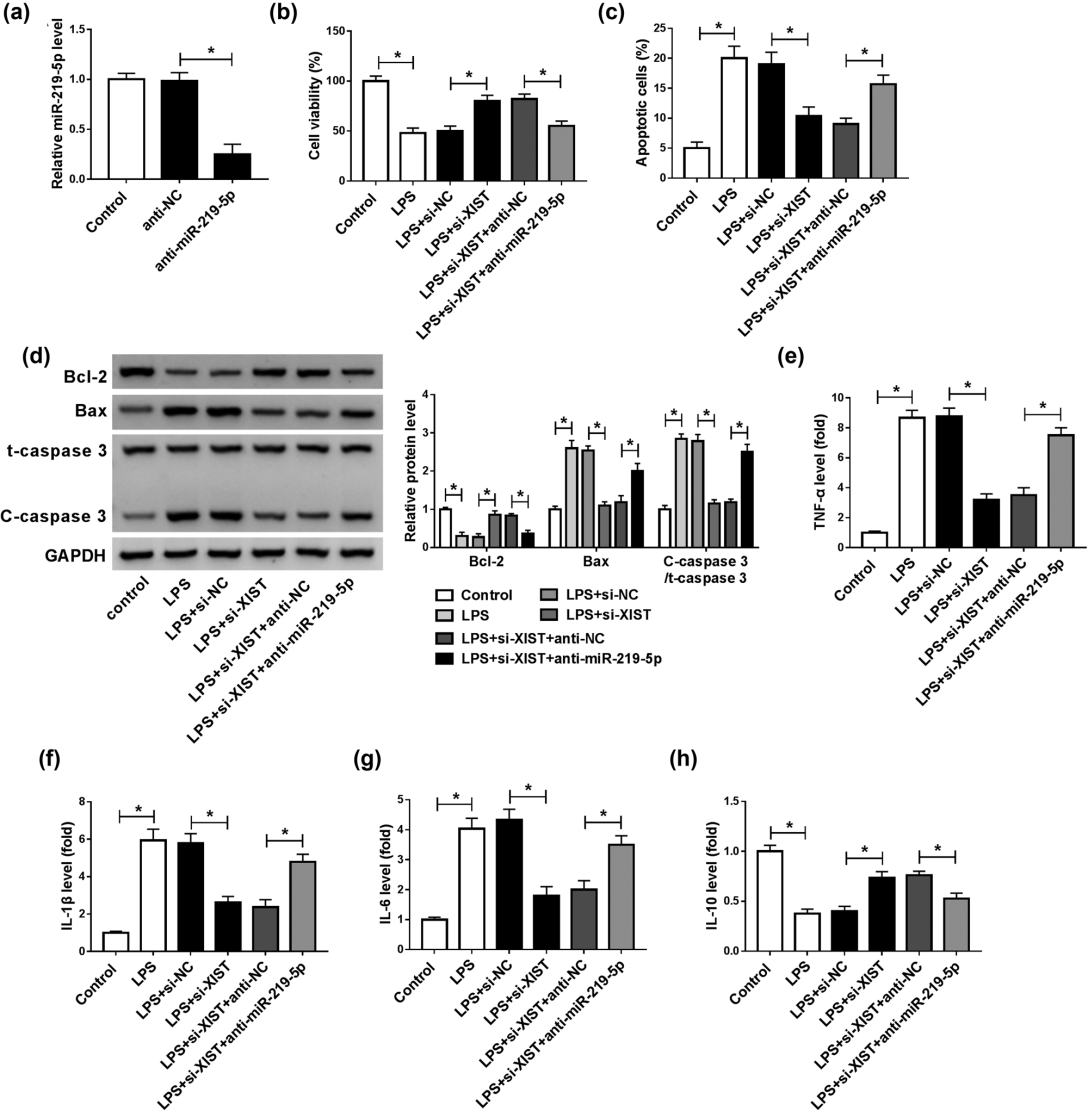
XIST knockdown regulated LPS-induced BV2 cell viability, apoptosis, and inflammatory response by interacting with miR-219-5p. (a) The expression of miR-219-5p in untransfected BV2 cells and anti-NC or anti-miR-219-5p transfected BV2 cells was determined using RT-qPCR assay. (b–h) BV2 cells were divided into six groups: control, LPS, LPS + si-NC, LPS + si-XIST, LPS + si-XIST + anti-NC, and LPS + si-XIST + anti-miR-219-5p. (b and c) BV2 cell viability and apoptosis were determined by CCK-8 assay and flow cytometry analysis, respectively. (d) The protein level of Bcl-2, Bax, t-caspase 3, and C-caspase 3 in BV2 cells were measured by western blot assay. (e–h) The levels of TNF-α, IL-1β, IL-6, and IL-10 in BV2 cells were detected by ELISA kits. *P < 0.05.

Subsequently, we further explored whether XIST could alter LPS-induced BV2 cell injury by targeting miR-219-5p. First, anti-miR-219-5p transfection evidently reduced the level of miR-219-5p in BV2 cells compared to anti-NC and control groups, indicating that anti-miR-219-5p was successfully transfected into BV2 cells (Figure 5a). Next, BV2 cells were assigned to control, LPS, LPS + si-NC, LPS + si-XIST, LPS + si-XIST + anti-NC, and LPS + si-XIST + anti-miR-219-5p groups. The results of the CCK-8 assay and flow cytometry analysis indicated that XIST knockdown promoted cell viability and inhibited apoptosis in LPS-stimulated BV2 cells, while the impacts were partially overturned by decreasing miR-219-5p (Figure 5b and c). Western blot assay showed that the promotional role in Bcl-2 level and the suppressive roles in Bax and C-caspase 3 levels mediated by XIST silencing in LPS-treated BV2 cells were ameliorated following the suppression of miR-219-5p (Figure 5d). In addition, ELISA results showed that the impacts of XIST deficiency on TNF-α, IL-1β, IL-6, and IL-10 levels were all restored by decreasing miR-219-5p expression in LPS-activated BV2 cells (Figure 5e–h). These outcomes suggested that XIST knockdown attenuated LPS-induced BV2 cell injury by targeting miR-219-5p.

### 3.6 LPS-induced XIST promoted the activation of NF-κB pathway by regulating miR-219-5p

**Figure 6:**
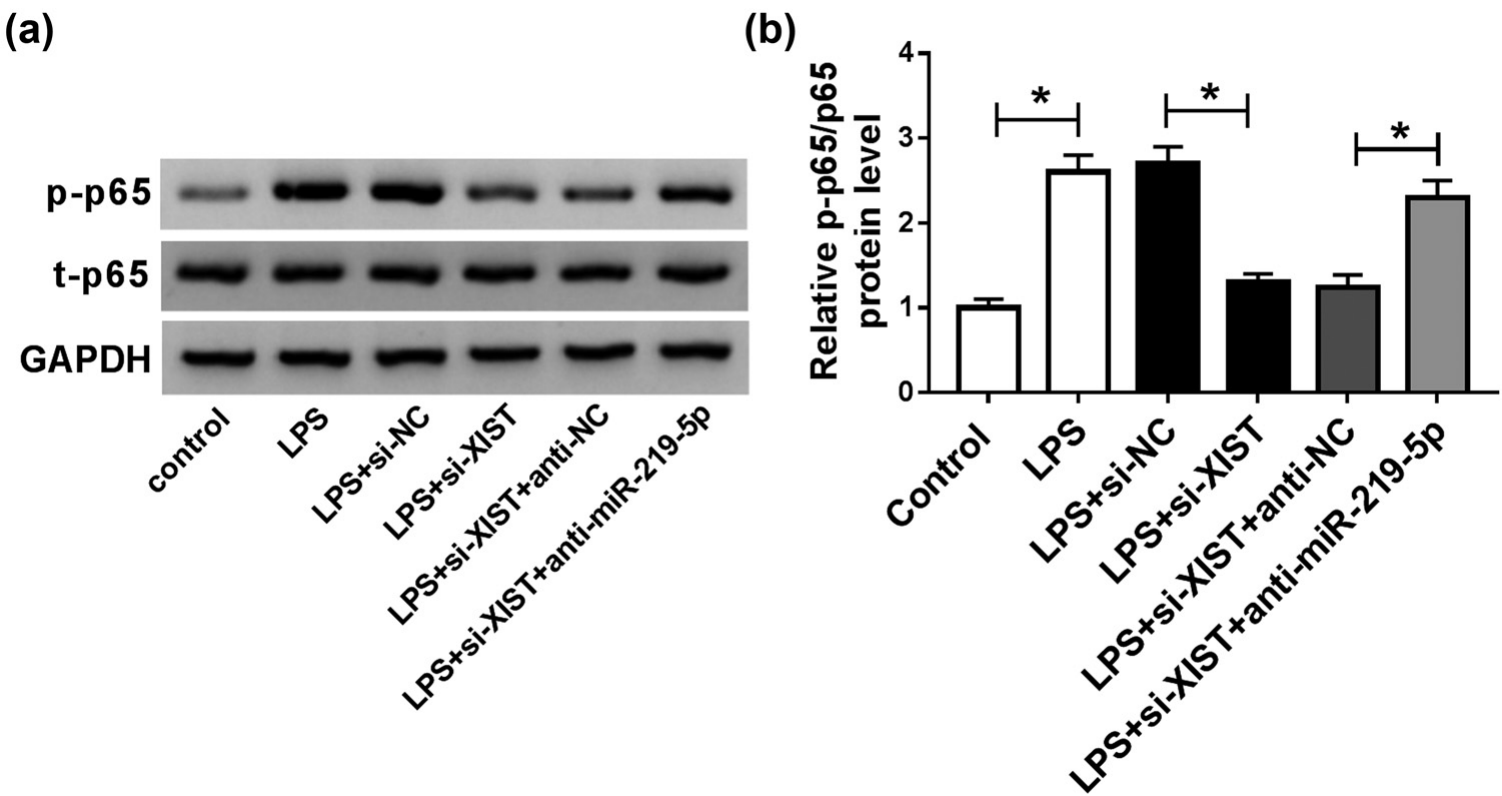
XIST silencing inactivated LPS-stimulated NF-κB pathway by targeting miR-219-5p. (a and b) BV2 cells were assigned to control, LPS, LPS + si-NC, LPS + si-XIST, LPS + si-XIST + anti-NC, and LPS + si-XIST + anti-miR-219-5p groups, and then, the protein levels of p-p65 and t-p65 were measured by western blot assay. *P < 0.05.

Finally, BV2 cells were divided into six groups: control, LPS, LPS + si-NC, LPS + si-XIST, LPS + si-XIST + anti-NC, and LPS + si-XIST + anti-miR-219-5p to explore the effect of XIST on LPS-mediated NF-κB signaling pathway activation in BV2 cells. As demonstrated by western blot assay, LPS treatment enhanced the protein level of p-p65 in BV2 cells, indicating the activation of the NF-κB pathway. Moreover, we found that XIST deficiency suppressed LPS-induced NF-κB pathway activation, as shown by downregulation of p-p65 protein level, while the effect was alleviated by the inhibition of miR-219-5p (Figure 6a and b). Taken together, XIST knockdown could inhibit LPS-activated NF-κB signaling pathway by modulating miR-219-5p expression in BV2 cells.

## 4 Discussion

lncRNAs have been proved as essential mediators in the development of SCI. After SCI, countless cytokines and signaling pathways have been demonstrated to mediate the apoptosis and inflammatory response [14,15]. LPS-stimulated microglial cells are widely utilized to explore the pathogenesis of SCI. In this study, we successfully constructed the SCI mice model and found that XIST was drastically increased in the spinal cord tissues of SCI mice. Moreover, the SCI cell model was constructed by stimulating BV2 cells with LPS. Then, we tested cell viability, apoptosis, and the levels of inflammatory cytokines in LPS-triggered BV2 cells. We found that cell viability was repressed and cell apoptosis and inflammatory response were induced, indicating the successful construction of the SCI cell model. Thereafter, we explored the functions and mechanisms of XIST in SCI development. As a result, XIST knockdown recovered LPS-stimulated BV2 cell injury by regulating miR-219-5p and NF-κB signaling pathway.

In the past decades, the potential functions of XIST in SCI have been gradually studied. For example, Kwon et al. revealed that the XIST level was enhanced in the SCI rat model [11]. Gu et al. manifested that XIST knockdown effectively limited the apoptosis of neuronal in SCI rats by modulating miR-494/phosphatase and tensin homolog deleted on chromosome ten (PTEN)/phosphoinositide 3-kinase (PI3K)/AKT [5]. Moreover, Zhao et al. reported that XIST silencing restored the suppressive role in cell viability and the promotional role in apoptosis and inflammation mediated by LPS in microglial cells by regulating miR-27a/smad ubiquitination regulatory factor 1 (Smurf1) axis [29]. Correspondingly, our results showed that XIST was conspicuously increased in LPS-triggered BV2 cells. XIST interference enhanced cell viability and impeded apoptosis, concomitant with upregulation in Bcl-2 level and downregulation in Bax and C-caspase-3 levels in LPS-triggered BV2 cells. In addition, our results exhibited that XIST knockdown reduced TNF-α, IL-1β, and IL-6 levels and enhanced IL-10 level in LPS-activated BV2 cells, suggesting that XIST deficiency attenuated LPS-induced inflammatory response in BV2 cells. Overall, XIST knockdown could accelerate the recovery of SCI through promoting microglial cell viability and impeding apoptosis and inflammation.

For mechanism analysis, the downstream target of XIST was investigated. XIST has been identified as the sponge for multiple miRNAs, such as miR-152 [26], miR-101 [3], miR-367 [16], and miR-137 [27]. While in our study, miR-219-5p was proved to be a target of XIST. It has been reported that miR-219-5p was downregulated in SCI mice [17]. Moreover, miR-219-5p was found to ameliorate inflammatory injury in the SCI mice model [32]. Herein, we observed that miR-219-5p inhibition abrogated the impacts of XIST knockdown on cell viability, apoptosis, and inflammation in LPS-activated BV2 cells, indicating XIST knockdown could attenuate SCI by targeting miR-219-5p.

It has been documented that the activation of the NF-κB pathway can trigger the production of pro-inflammatory cytokines, thereby inducing inflammatory response and apoptotic response [12,13,20]. Moreover, NF-κB pathway activation plays a positive role in the SCI development [2,10]. For example, GRB1 relieved SCI via altering miR-130b-5p/TLR4/NF-κB pathway [23]. circ_0000962 inhibited the inflammation in the SCI cell model via activating PI3K/Akt and inactivating NF-κB by sponging miR-302b-3p [7]. Thus, we explored the impact of LPS-induced XIST in the NF-κB pathway. Our results showed that the knockdown of XIST decreased LPS-induced p-p65 level in BV2 cells, while miR-219-5p suppression restored the effect, suggesting that XIST silencing might block LPS-stimulated NF-κB pathway by targeting miR-219-5p.

In conclusion, this study uncovered that XIST was upregulated in SCI mice and LPS-activated BV2 cells. XIST knockdown ameliorated LPS-induced microglial cell apoptosis and inflammatory injury after SCI by sponging miR-219-5p and inactivating NF-κB pathway. Our study revealed the protective effect of XIST silencing in SCI, which deepened our understanding on the molecular basis in the management of SCI and might provide a novel direction for SCI therapy.
